# Breeding records and the detection of nesting predators of wild‐release red‐crowned cranes into non‐breeding areas of the Yancheng National Nature Reserve, China

**DOI:** 10.1002/ece3.11322

**Published:** 2024-04-22

**Authors:** Wu Dawei, Hu Xinyi, Chen Hao, Chen Guoyuang, Chen Weihua, Lu Changhu

**Affiliations:** ^1^ College of Life Sciences Nanjing Forestry University Nanjing China; ^2^ Yancheng National Nature Reserve for Rare Birds Administrative Bureau Yancheng China

**Keywords:** breeding habitat, nest predation, population restocking, red‐crowned cranes

## Abstract

The western population of the red‐crowned crane *Grus japonensis* in mainland China has been decreasing in the past few decades and wild population enhancement programmes have been launched in this country. First, 14 captive‐bred red‐crowned cranes were released into the core area of Yancheng National Nature Reserve for Rare Birds (YNNR), one of the most important wintering areas of this species, in 2013 (seven individuals) and 2015 (seven individuals) and then 8 more captive‐bred cranes were released into YNNR from February 2022 to February 2023. We used satellite positioning and drone monitoring to study the status of released cranes. The results showed that two individuals (No. BJZ001 and BJZ008) from the first group of released cranes were found breeding in 6 of 7 years in the YNNR from 2017 to 2023. Three individuals (No. WNNR022, WNNR025 and WNNR026) from the second group of released cranes were recorded breeding in YNNR in 2023. All released cranes lived in the YNNR year round and did not migrate with wild cranes. Raccoon dogs, *Nyctereutes procyonoides*, were first recorded as red‐crowned crane nest predators in the YNNR. Although these released cranes breed successfully in the YNNR, nestlings might face the threat of predators in non‐traditional breeding areas. Further research is needed to determine whether offsprings of released individuals migrate with wild cranes and if breeding in their original wintering grounds is truly beneficial for population growth.

## Graphical abstract

We continue to track the breeding of wild‐release red‐crowned cranes in wintering grounds and for the first time we have found nest predators, Raccoon dog.

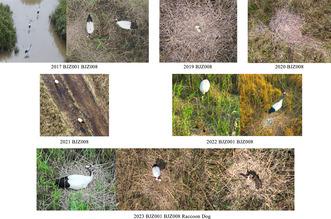

## 1 INTRODUCTION

Human activities have led to environmental isolation and habitat fragmentation. It poses a serious threat to the survival and reproduction of endangered wildlife. This leads to the extinction of species and the depletion of biodiversity (Daskin & Pringle, 2018; Haddad et al., 2015). A viable approach to conserving endangered species and restoring long‐term self‐sustaining wild populations is through artificial intervention, such as introduction, reintroduction and restocking (Pritpal & Philip, 1998). Restocking refers to the intentional release of many organisms into the habitat of an existing population to increase the population size (IUCN, 1987). Recently, China has witnessed successful population recovery cases of species such as the Hainan gibbon *Nomascus hainanus* and the crested ibis *Nipponia nippon* (Chen et al., 2013; Li et al., 2010).

The red‐crowned crane *Grus japonensis* is one of the 15 existing crane species. It is primarily distributed in East Asia and is classified as a national first‐class protected animal in China. In 2021, it was listed as Vulnerable (VU) by the International Union for Conservation of Nature (IUCN) (BirdLife International, 2021). Recent reports from partners compiled via the International Crane Foundation suggest that the total global population may be closer to 4150. As a large waterbird relying on wetlands for survival, the red‐crowned crane occupies the top trophic level in the wetland ecosystem food chain and serves as a bioindicator species for the dynamic changes in wetland environments (Wu et al., 2016; Zhang et al., 2019). According to previous research, the known wild populations of red‐crowned cranes can be classified into island populations and mainland populations, with the island populations being resident birds that are exclusively found in Hokkaido, Japan (Su & Zhou, 2012). The mainland population is divided into two parts, namely, the eastern and western populations. The western population breeds in the middle reaches of the Heilongjiang River on its Russian side, as well as in the Songnen Plain and Da Hinggan Mountains region. They primarily winter in the coastal wetlands of Yancheng, Jiangsu Province (Zhang et al., 2019; Zhou et al., 2021). According to survey findings, this population of the species is gradually declining. Habitat alteration caused by anthropogenic disturbances is considered one of the primary factors contributing to the decline in this particular population (Wang et al., 2020).

The reintroduction of the red‐crowned crane in the wild is an effective measure to enhance its wild population. According to statistics, there are 2386 captive red‐crowned cranes in China, including 1176 individuals in zoos and 1210 in nature reserves (Lin et al., 2021). From late November to early December 2013, management personnel released a total of seven captively‐bred red‐crowned cranes hatched under natural conditions into the core area of the Yancheng Wetland Rare Bird National Nature Reserve in Jiangsu Province and then another seven were released in July 2015 (Hua et al., 2018; Xu et al., 2020). In 2017, a total of two individuals (designated BJZ001 and BJZ008) of the aforementioned captively‐bred Siberian cranes successfully formed pairs in the wild in Yancheng, China. Moreover, they successfully built nests and laid eggs, resulting in the hatching of two crane chicks (Hua et al., 2018).

## 2 LOCATION AND METHODS

### 2.1 Observation location

**FIGURE 1:**
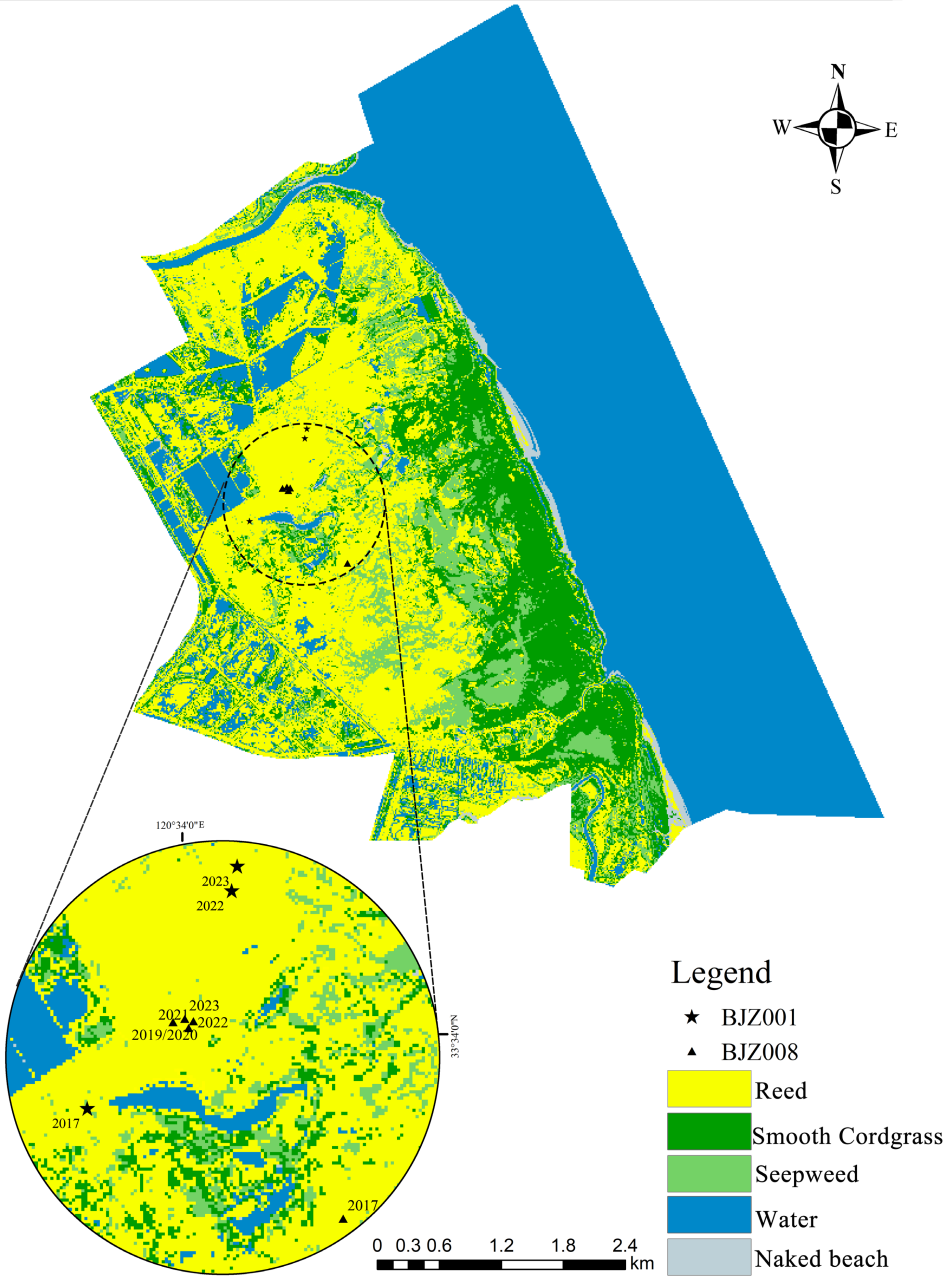
Land‐use classification and annual nest sites of red‐crowned crane breeding sites in the core area of the protected area.

The Yancheng National Nature Reserve is located in the eastern coastal area of China (32°48′–34°29′ N, 119°53′–121°18′ E). It possesses unique mudflat coastal zones and various tidal wetland ecosystems, providing important habitats for wetland birds and serving as one of the wintering habitats for wild red‐crowned cranes (Wang et al., 2020; Xu et al., 2020). The vegetation communities along the coastal zone of the core area are distributed in a stratified pattern, ranging from the coastline itself to the exposed tidal flats and further inland. These layers are interlaced with species such as the smooth cordgrass *Spartina alterniflora*, the seepweed *Suaeda salsa* and the reed *Phragmites australis* (Li et al., 2019) (see Figure 1).

### 2.2 Methods

In the early stage of the study, the red‐crowned cranes released into the YNNR were all individuals bred in captivity in zoos. They were equipped with satellite positioning signal transmitters (Global Messenger, solar‐powered, 15 g) during the release to monitor and record their activity trajectories. From November 2022 to March 2023, the nature reserve team initiated the release of more captive‐bred red‐crowned cranes. This release mainly focused on pairs of mates or kin cranes, totalling seven individuals (with identification numbers WNNR017, WNNR018, WNNR20‐22, WNNR25, WNNR26; see Table 1). These cranes were equipped with satellite transmitters (Global Messenger) attached to leg bands for monitoring and recording their activity trajectories. The activing situation of the wild‐release red‐crowned cranes was observed through Nikon Z9 camera (600×) and monocular telescope (Magnification 20–60×). The frequency of observation is once a month. When we find that wild‐release red‐crowned cranes have reproductive behaviour, we used the drones (DJI UAV, Mavic 2) to reach about 100 m above the breeding cranes to shoot. Red‐crowned cranes is alert to this behaviour. In order to prevent excessive interference, we only conduct an unmanned observation once a month.

**TABLE 1:** Information about wild‐release crane in Yancheng.

Number	Date of releasing	Sex	Age
WNNR017	2022.02.25	Male	5
WNNR018	2022.02.25	Female	5
WNNR020	2022.11.03	Unknown	0.5
WNNR021	2022.11.03	Unknown	0.5
WNNR022	2022.11.03	Female	8
WNNR025	2023.02.16	Female	10
WNNR026	2023.02.16	Male	10

## 3 RESULTS

BJZ008 and BJZ001 were release on 9 January and 10 January, 2015 (Cui et al., 2017). Successfully hatched a chick each in the reed marsh on 6 May 2017 and 4 June 2017, respectively. In late November, observations were made of the juvenile birds actively following their parents. With the exception of the year 2018, these female birds have been recorded breeding thereafter (see Table 2).

**TABLE 2:** Historical breeding records of *Grus japonensis* (BJZ001 and BJZ008) in Yancheng.

Year	Number	Date of filming	Latitude	Longitude	Nesting behaviour	Lays eggs	Incubates	Predators
2017	BJZ001	6.4	33.55948	120.5575	Y	Y	Y	
	BJZ008	5.6	33.55036	120.5844	Y	Y	Y	
2019	BJZ001							
	BJZ008	3.29	33.56718	120.5662	Y	Y	N	
2020	BJZ001							
	BJZ008	4.27	33.56718	120.5662	Y	N	N	
2021	BJZ001							
	BJZ008	4.19	33.5667	120.5679	Y	Y	Y	
2022	BJZ001	5.17	33.57878	120.572	Y	Y	Y	
	BJZ008	4.26	33.5673	120.5683	Y	Y	N	
2023	BJZ001	5.11	33.58094	120.5725	Y	Y	N	
	BJZ008	4.14	33.56749	120.5674	Y	Y	N	Y

**FIGURE 2:**
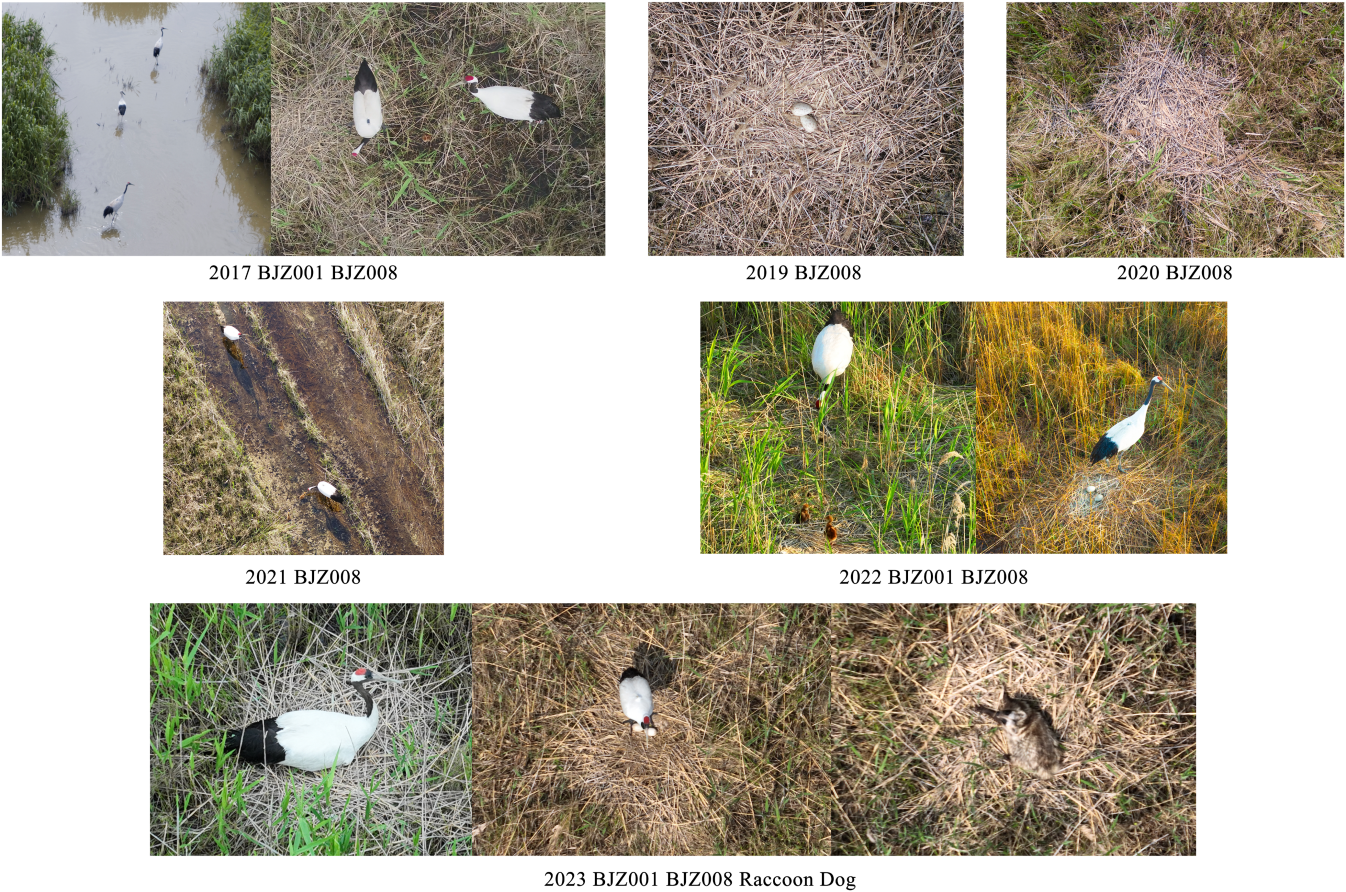
The reproductive records and detection of nest predators for BJZ001 and BJZ008 monitored by unmanned aerial vehicles.

On 14 April 2023, the nesting behaviour of BJZ008 was documented again through drone monitoring. On 12 May 2023, a second‐level protected animal, a raccoon dog *Nyctereutes procyonoides*, was captured on a camera preying on the breeding nest (see Figure 2).

**FIGURE 3:**
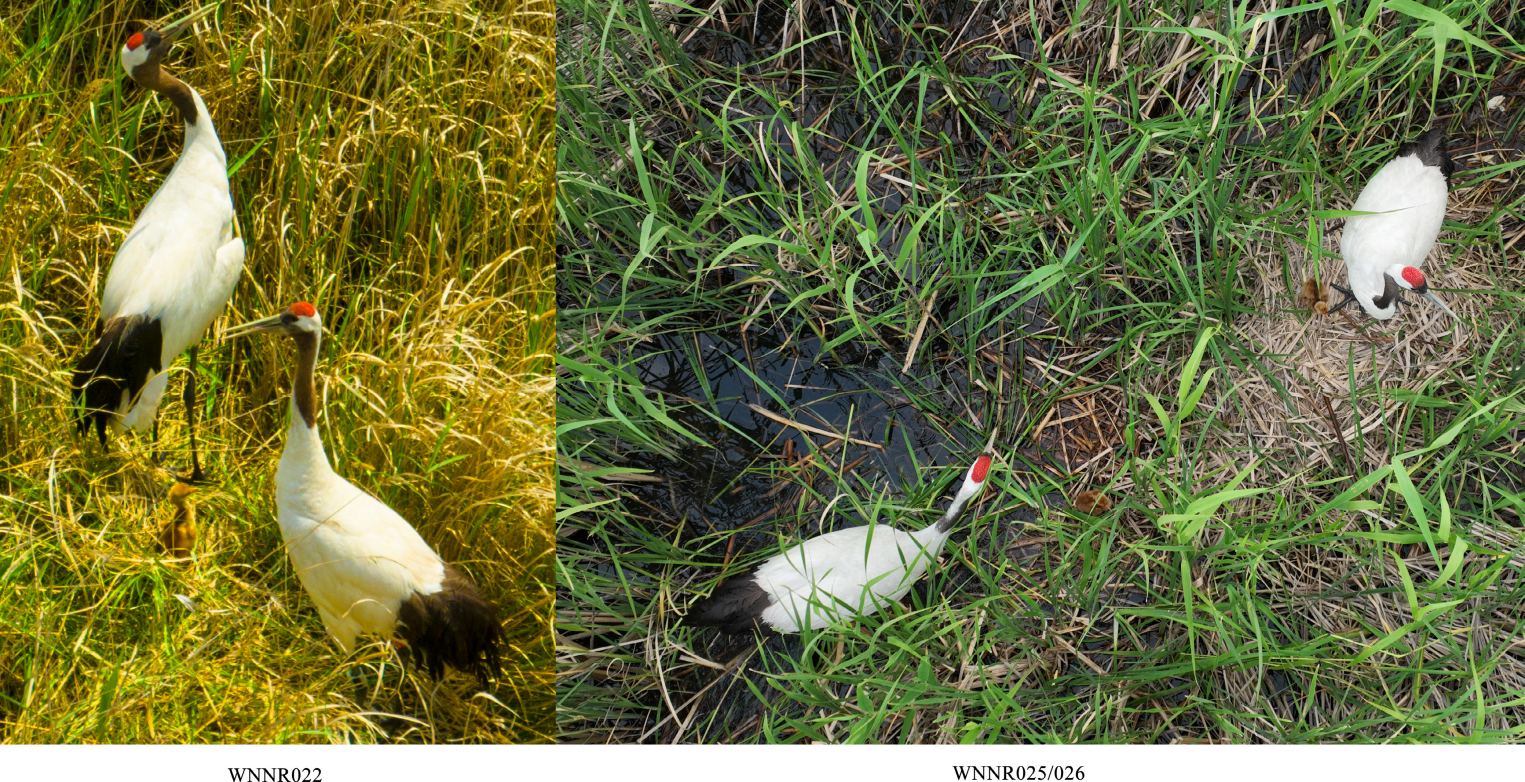
Drone‐monitored breeding records in 2023 (WNNR022/025/026).

Among the red‐crowned cranes released into the reserve, three individuals have been tracked for breeding since 2023. Two of them, WNNR025 and WNNR026, were already paired before release, while the third one, WNNR022, paired and bred in the wild with an injured crane unable to migrate (see Figure 3).

## 4 DISCUSSION

The wild eastern population of red‐crowned cranes has shown an increasing trend in numbers over the years, while the western population, on the contrary, has shown a decreasing trend. Captive breeding and subsequent release into the wild can play a significant role in the population growth process (Yoo et al., 2022; Zhou et al., 2016, 2021). Compared to animals bred in zoos, the population of this species in natural reserves is larger and their living environment is better. For example, there are more natural habitats and less human interference, which helps to improve the success rate of breeding compared to that in captivity (Zhou et al., 2016). The Whooping Crane was once listed as an endangered species same as red‐crowned crane. After extensive discussions by experts, in 2011, 10 juvenile Whooping Cranes arrived at White Lake in southwestern Louisiana and began establishing a non‐migratory population (Gomez, 2014). Previously, a migratory population was successfully established in the eastern United States in the fall by ultralight plane and direct autumn release (Urbanek et al., 2010, 2014). As a result, the population of Whooping Cranes has increased. Two tracked cranes, who were bred in zoos successfully, paired after being released into natural reserves. In addition, there has been a breeding record every year since 2018 for the cranes released into the reserve. However, unlike wild red‐crowned cranes, the released red‐crowned cranes do not migrate to traditional breeding grounds with other wild crane populations but instead breed locally in the wintering area. We speculate that there may be tworeasons for this phenomenon. First, the habitat conditions within the core area of the Yancheng Nature Reserve are similar to those of the main breeding ground (e.g., Zhalong Nature Reserve), both being large areas of reed marshes (Gao et al., 2021), providing abundant habitat and food resources. Second, the two artificially hatched zoo‐reared cranes were directly released to the wintering area during the wintering period. Although they underwent wilding training prior to their release, they still were not able to adapt to migratory activity. The successful reintroduction of the Whooping Crane in America provides us with valuable reference.

Observations on the resident population of red‐crowned cranes in Hokkaido, Japan, have revealed that the island population of red‐crowned cranes utilises fixed breeding grounds annually (Masatomi, 2000). A pair of cranes collectively guards a breeding territory ranging from 1 to 12.3 square kilometres (Kitagawa, 1982; Viniter, 1981). Satellite tracking data indicate that the breeding nest site of the stable breeding female crane BJZ008 is relatively concentrated, with two consecutive years of using the same nest (in 2019 and 2020). However, overall, the breeding ground selection of the two female cranes is not fixed and we speculate that this may be due to the presence of nest predators that cannot be resisted locally. In 2023, we also captured footage for the first time, through drone monitoring, showing a local nest predator, the raccoon dog, lying on the positions where two crane eggs were originally located.

As the population of red‐crowned cranes continues to recover, the number of red‐crowned cranes released into the YNNR gradually continues to increase. Despite multiple breeding records, the proportion of breeding cranes among the released population remains low and the breeding success rate is very low. However, it can be confirmed that with the increasing number of released red‐crowned cranes, the YNNR has gradually transformed from an exclusively wintering site to a breeding and wintering site. It is currently unclear whether the offspring of released cranes migrate with wild cranes, but at this stage of the process, managers still need to prioritise new challenges such as nest predators.

## AUTHOR CONTRIBUTIONS

**Wu Dawei:** Data curation (equal); validation (lead); visualization (equal); writing – original draft (lead); writing – review and editing (equal). **Hu Xinyi:** Data curation (equal); visualization (equal); writing – review and editing (equal). **Chen Hao:** Data curation (lead); project administration (lead). **Chen Guoyuang:** Investigation (equal); resources (equal). **Chen Weihua:** Investigation (equal); resources (equal). **Lu Changhu:** Conceptualization (lead); funding acquisition (lead); project administration (lead); writing – review and editing (lead).

## CONFLICT OF INTEREST STATEMENT

The authors have no conflicts of interest to declare.

## Data Availability

Data sharing is not applicable to this article because no new data were created or analysed in this study.
